# The Complex Interplay between Toxic Hallmark Proteins, Calmodulin-Binding Proteins, Ion Channels, and Receptors Involved in Calcium Dyshomeostasis in Neurodegeneration

**DOI:** 10.3390/biom14020173

**Published:** 2024-01-31

**Authors:** Danton H. O’Day

**Affiliations:** 1 Department of Biology, University of Toronto Mississauga, Mississauga, ON L5L 1C6, Canada; danton.oday@utoronto.ca; 2 Cell and Systems Biology, University of Toronto, Toronto, ON M5S 3G5, Canada

**Keywords:** calcium dysregulation, neurodegeneration, ion channels, calmodulin-binding proteins, amyloid beta, Tau, alpha-synuclein, huntingtin, Calmodulin Hypothesis

## Abstract

Calcium dyshomeostasis is an early critical event in neurodegeneration as exemplified by Alzheimer’s (AD), Huntington’s (HD) and Parkinson’s (PD) diseases. Neuronal calcium homeostasis is maintained by a diversity of ion channels, buffers, calcium-binding protein effectors, and intracellular storage in the endoplasmic reticulum, mitochondria, and lysosomes. The function of these components and compartments is impacted by the toxic hallmark proteins of AD (amyloid beta and Tau), HD (huntingtin) and PD (alpha-synuclein) as well as by interactions with downstream calcium-binding proteins, especially calmodulin. Each of the toxic hallmark proteins (amyloid beta, Tau, huntingtin, and alpha-synuclein) binds to calmodulin. Multiple channels and receptors involved in calcium homeostasis and dysregulation also bind to and are regulated by calmodulin. The primary goal of this review is to show the complexity of these interactions and how they can impact research and the search for therapies. A secondary goal is to suggest that therapeutic targets downstream from calcium dyshomeostasis may offer greater opportunities for success.

## 1. Introduction

The importance of calcium homeostasis in the function of neurons is well-established [1,2,3]. Calcium signaling mediates multiple critical neuronal events including exocytosis, gene regulation, plasticity, synaptic transmission, and synaptogenesis, among others. Thus, disrupting calcium levels can affect all of these critical events. A common theme for neurodegenerative diseases is the dysregulation of cytosolic calcium levels [4,5,6,7,8]. The disruption of calcium signaling and its role in neurodegeneration and neuronal death has been well-studied in Alzheimer’s disease (AD), Huntington’s disease (HD), and Parkinson’s disease (PD) [6,7,9].

AD, HD, and PD are major neurodegenerative diseases that are associated with brain pathologies involving disease-linked toxic proteins and their aggregates [10,11,12]. Each of these disorders has a different age of onset, clinical symptoms, and pathological features that affect different neurons. While aging itself does not cause them, like many other neurodegenerative diseases, the chance of developing one of them increases significantly with age. Initially, neurodegeneration is evidenced by multiple psychological issues (e.g., cognitive and emotional disorders; altered social behaviors) that can ultimately progress to anxiety, bipolar disorder and schizophrenia as dementia sets in [13]. These three diseases have well-defined toxic hallmark proteins that are intimately associated with their neuropathology: AD (amyloid beta, Aβ; phosphorylated Tau, pTau); HD (mutant huntingtin, mHtt); PD (α-synuclein, αSyn) [12].

**Figure 1:**
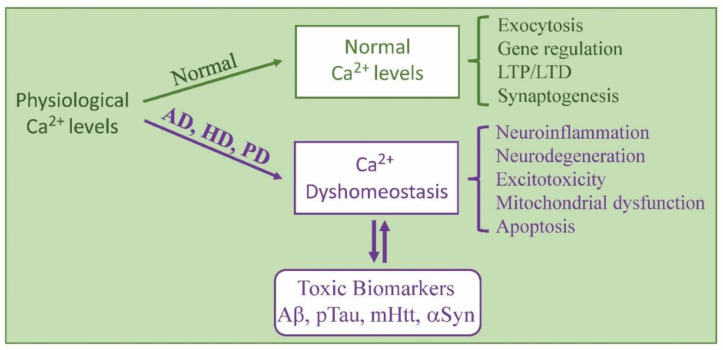
A summary of calcium-dyshomeostasis-related neurodegenerative events in Alzheimer’s (AD), Huntington’s (HD), and Parkinson’s (PD) diseases. Normal physiological levels of intracellular calcium regulate essential neuronal functions including exocytosis, gene regulation, and other events. Calcium dyshomeostasis is an early event in AD, HD, and PD that is involved in the production of disease-related toxic biomarkers (amyloid beta, Aβ; phosphorylated Tau, pTau; mutant huntingtin protein, mHtt; alpha-synuclein, αSyn). Each of these toxic biomarkers in turn adds to calcium dyshomeostasis by affecting receptor and ion channel function. Events linked to the disruption of calcium levels include neuroinflammation, mitochondrial dysfunction, and others, some of which also feed back to compound dysregulated calcium levels.

Evidence continues to be revealed indicating that the early disruption of calcium homeostasis interferes with neuronal events that in turn feed back to further disrupt calcium levels and calcium-mediated signal transduction (Figure 1). For example, as detailed below, Aβ, pTau, mHtt, and αSyn can each feed back, causing persistent calcium dysregulation affecting downstream calcium-binding proteins (CaBPs) including calpain and calmodulin (CaM) that, in turn, also affect calcium levels in the cytosol, endoplasmic reticulum, mitochondria, and lysosomes. After a brief introduction to calcium homeostasis and dyshomeostasis and its effects on CaBPs, each of the three primary diseases covered in this review and the general impact of calcium dysregulation on them are overviewed. After that, specific ion channels and receptors linked to neurodegenerative events are discussed. The goal here is not to review the specific functions of each receptor and channel but instead to first show their importance in neurodegeneration and second to reveal that they interact with a number of disease-linked proteins that potentially affect their functions.

### 1.1. Receptors and Ion Channels Involved in Calcium Homeostasis and Dyshomeostasis

**Figure 2:**
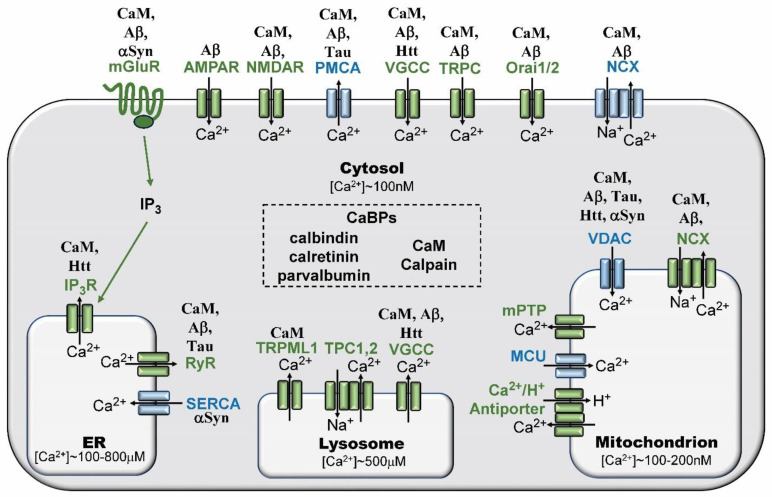
A diagrammatic summary of receptors, ion channels, sensors/effectors, and buffering proteins involved in calcium homeostasis and dysregulation and their binding to toxic biomarkers and calmodulin. Calcium influx (green channels) into the cytoplasm occurs across the cell membrane through multiple channels (AMPAR, NMDAR, VGCC, TRPC, and Orai1/2). Cytoplasmic contributions to increased intracellular calcium levels come from the endoplasmic reticulum (ER: IP3R and RYR), mitochondria (NCX, mPTP, and Ca^2+^/H^+^ antiporter) and lysosomes (TPC1,2, TRPML1, and VGCC). Intracellular calcium levels can be reduced by efflux (blue channels) via the cell membrane (NCX and PMCA) or by uptake into the ER (SERCA) and mitochondria (MCU, VDAC). Interactions of calmodulin (CaM) and individually studied toxic proteins (Aβ, Tau, Htt, and αSyn) with receptors and ion channels are indicated. The specific receptor and channel subtypes that interact with them are covered in the main text. The acronyms used in the figure are covered in the main text and listed in the Abbreviations.

The precise regulation of cytosolic free calcium levels is controlled by a diversity of receptors and ion channels in the cell membrane, in cytoplasmic compartments (i.e., endoplasmic reticulum, lysosomes, mitochondria), and by various CaBPs that serve as buffers and effectors (Figure 2) [14,15]. Extracellular levels of free calcium are in the millimolar range (1.1–1.4 mM), but the normal, cytosolic calcium concentration in neurons and other cell types is maintained around 100 nM. A complex interplay ensures that cytosolic calcium homeostasis is carefully controlled. When it is not, the impact can be devastating. Calcium dysregulation can result from toxic peptides forming unregulated cell membrane pores, altered regulation of cell membrane receptor channels, altered release from intracellular stores, changes in buffering protein levels or function, and by the excessive increase in extracellular calcium caused by the death of adjacent neurons.

As summarized in Figure 2 and detailed below, many receptors and channels involved in calcium homeostasis and dysregulation bind to CaM: mGluR (metabotropic glutamate receptor), NMDAR (N-methyl-D-aspartate receptor), PMCA (plasma membrane Ca^2+^-ATPase), VGCC, (voltage-gated calcium channel), Orai1/2, (calcium-release-activated calcium channel protein 1 and 2), NCX (Na^+^/Ca^2+^ exchanger), VDAC (voltage-dependent anion channel 1), IP3R (inositol (1,4,5)-trisphosphate receptor), RyR (ryanodine receptor), and TRPML (TRP ion channel family) [16,17,18,19,20,21,22,23,24,25,26]. In addition to binding to CaM, a number of these receptors and channels bind to toxic protein biomarkers (Aβ; Tau; Htt; aSyn) linked to specific neurodegenerative diseases: mGluR5 (Aβ; αSyn), NMDAR (Aβ), PMCA (Aβ; Tau), VGCC (Aβ; Htt), TRPC (Aβ), Orai (Aβ), NCX (Aβ), VDAC (Aβ; Tau; Htt; αSyn), IP3R (Aβ; Htt), RyR (Htt) [17,18,22,24,27,28,29,30,31,32,33,34,35,36,37,38]. Two other ion channels do not bind CaM, but each one binds to a specific toxic marker: AMPAR (Aβ) and SERCA (αSyn) [39,40]. It should also be noted, as covered in more detail below, that four toxic markers (Aβ; Tau; Htt; αSyn) each also bind to CaM [41,42,43,44].

### 1.2. Calcium-Binding Proteins: Calpain and Calmodulin

To understand the impact of calcium dysregulation, it is important to look downstream at CaBPs, especially CaM, since it is a primary regulator of the receptors and ion channels involved in calcium homeostasis and dysregulation (Figure 2). Most free cytosolic calcium is bound to CaBPs, of which some serve as buffers (e.g., calbindin, calretinin, parvalbumin) and others as effectors (e.g., CaM, calpain). Due to their buffering abilities, CaBPs serve as a first line of defense against the toxic dysregulation of calcium ions [45]. Although there are hundreds of CaBPs, only a handful have been well-studied (e.g., CaM, calcium-activated neutral proteases (esp. calpains), calbindin, calretinin and parvalbumin). Each of these is an EF-hand CaBP and all are expressed in hippocampal neurons as well as differentially within other brain regions. The three proteins that are primarily recognized as calcium buffers (calbindin, calretinin, and parvalbumin) decrease in expression levels with age, and gene knockouts for each of them impact synaptic plasticity in various ways [46]. Their importance in buffering high calcium levels is reinforced by their high-capacity calcium binding (parvalbumin, (Kd 9 nM), calbindin (Kd 393 nM), calretinin (Kd 1.5 μM)), but their value as therapeutic targets remains in question [45,47]. A protective neuronal protein involved in calcium homeostasis, calbindin D undergoes a decrease in AD brains coupled with an increase in CSF levels that correlates with p181Tau levels, but its value as a diagnostic tool or therapeutic target requires further study [47].

A number of studies have examined the functions of calpain in neurodegeneration. There are multiple isoforms of the calcium-activated neutral protease calpains. Calpains 1 and 2 have opposing functions. In normal brains, calpain 1 is neuroprotective, functioning in certain types of synaptic plasticity and related examples of learning and memory. In contrast, calpain 2 is neurodegenerative since it is linked to cell death and counteracts plasticity and learning. These attributes and others are reviewed in Wang et al. (2020) [48]. Calpains serve multiple roles in neurodegenerative events including cleaving and activating protein kinase Cα and glycogen synthase kinase 3β, affecting αSyn aggregation and cleaving critical proteins involved in normal neuronal functions [48,49]. As another example, multiple and diverse lines of evidence argue that an unregulated increase in neuronal calcium levels is central to the onset and progression of HD, and this increased calcium activates calpain, which cleaves mHtt to generate smaller toxic peptide fragments and increase αSyn aggregation, an event discussed below [49,50].

### 1.3. Calmodulin and Calmodulin-Binding Proteins

**Figure 3:**
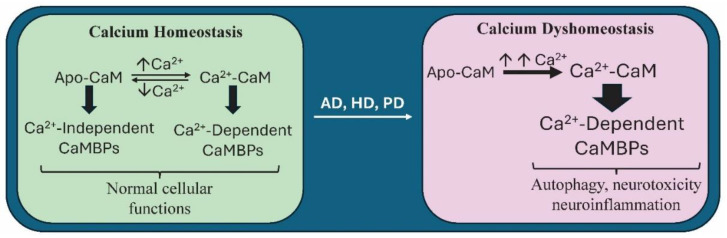
A comparison of the fundamental effects of calcium homeostasis versus calcium dyshomeostasis on calcium calmodulin function. At normal physiological levels of calcium, altered levels of the divalent cation can shift calmodulin (CaM) from calcium-free CaM (apoCaM) that binds to calcium-independent calmodulin-binding proteins (CaMBPs) to a calcium-bound CaM (Ca^2+^-CaM) that binds to calcium-dependent CaMBPs. The balance between these events controls normal cellular functions. As described by the Calmodulin Hypothesis, calcium dyshomeostasis in neurodegenerative diseases including Alzheimer’s (AD), Huntington’s (HD), and Parkinson’s (PD) can result in high levels of calcium over-activating Ca^2+^-CaM that in turn drives abnormal activities of calcium-dependent CaMBPs, leading to events including, but not limited to, autophagy, neurotoxicity, and neuroinflammation.

Since calcium dysregulation is an early event in neurodegeneration in AD, HD, and PD, among others, and since CaM is a primary downstream calcium target and effector, it is important to understand CaM’s involvement since it could lead to new therapeutic targets [51]. CaM binds to hundreds of binding proteins (CaMBPs) and it is essential for the function and survival of all eukaryotic cells [52]. In neurons, CaM oversees multiple critical functions: neurotransmission, exocytosis, ion channel function, synaptic strength, and plasticity, as well as learning and memory, among others (Figure 3). Calcium dyshomeostasis in neurodegenerative diseases shifts the calcium-mediated calmodulin signaling, disrupting these normal events and initiating harmful changes (Figure 3). To appreciate how this is possible, it is important to overview how CaM works.

CaM is a small (148aa; 16.7 kDa), highly conserved protein that is expressed at high levels in neurons. CaM’s binding ability is related to a flexible helix that joins the calcium-binding N- and C-terminal lobes. Calcium-free or apoCaM is a comparatively compact protein that upon calcium binding undergoes a marked conformational change into an extended configuration, exposing critical hydrophobic amino acids for CaMBP binding [53]. Unlike other protein regulators that bind to their targets with a relatively conserved binding sequence, CaM employs different binding domains (CaMBDs) in the absence and presence of calcium ions [53,54,55]. Calcium-free or apoCaM binds to CaMBPs via IQ ([FILV]Qxxx[RK]Gxxx[RK]xx[FILVWY]) motifs, IQ-like ([FILV]Qxxx[RK]Gxxxxxxxx) motifs, or unconventional IQ variants [53]. In the presence of calcium ions, Ca^2+^-CaM typically binds to CaMBPs predominantly via a variable sequence of hydrophobic amino acids, within an 18–22 stretch of amino acids, whose positions define canonical calcium-dependent binding motifs (e.g., 1-10, 1-5-10, 1-12, 1-8-14, 1-16) [53,54]. Continued research has revealed that, in addition, non-canonical binding occurs involving different sequence lengths, hydrophobic motifs, and unique moieties (e.g., myristoylated proteins) [54,56,57]. Additionally, differential calcium binding to the lobes and post-translational modifications to CaM (e.g., phosphorylation) that affect CaM function are still being studied [58]. Algorithms can reveal presumptive CaMBDs but, as with any theoretical domain, validation requires the analysis of deletion constructs and other molecular approaches [59]. Algorithmic predictions over the past 20-plus years have been relatively successful at defining CaMBDs, and the continued development of computational methods coupled with machine learning will undoubtedly improve CaMBP prediction [55]. Specific CaMBPs and, where identified, their CaMBDs associated with AD, HD, and PD are covered below in each disease-specific section.

As summarized in Figure 2 and discussed below, the toxic hallmark proteins for AD, PD, and HD (i.e., Aβ, Tau, αSyn, and Htt) bind to CaM. In addition, CaM binds to and differentially regulates a large number of receptors and calcium channel types (i.e., mGluR, NMDAR, PMCA, VGCC, TRPC, NCX, VDAC, IP3R, and RyR). CaMBDs have not been defined for all of these receptors and calcium channels, but several have been identified and reviewed [54]. Aβ, which binds to CaM, also binds to most of these receptors and channels (i.e., mGluR, NMDAR, PMCA, VGCC, TRPC, SOC, NCX, VDAC, and RyR), but it binds to only one non-CaM-binding channel (i.e., AMPAR). Two reviews discuss the binding of Aβ to AMPAR and its negative impact on channel function, especially as related to LTP and LTD [39,60]. αSyn, a CaMBP, binds to two Aβ/CaM-binding receptor channels (mGluR and NMDAR) as well as IP3R and SERCA. αSyn affects mGluR and calcium transit through NMDAR and AMPAR, contributing to intracellular toxic increases in calcium [61]. Htt interacts with two Aβ/CaM channels: VGCC and IP3R [62,63]. All IP3R subtypes (1–3) possess CaMBDs through which they bind CaM, but CaM’s inhibitory role in IP3R function remains to be elucidated [24]. The CaMBP Tau interacts with cell membrane PMCA and calcium channels in mitochondria (VDAC) and the ER (IP3R, RyR). Individually and in total, all of these multiple interactions add a level of complexity to the relevant diseases that complicates our understanding not only of calcium dysregulation but also the targeting of calcium ion channels as a therapeutic approach. The potential impact of such interactions is exemplified in the next section.

## 2. Calcium Regulation and Neurodegeneration

### 2.1. Alzheimer’s Disease

The major cause of dementia worldwide, Alzheimer’s disease (AD) is an age-related progressive neurodegenerative disease that is characterized by the loss of memory, impaired communication, depression, and other behavioral issues [64]. Historically it has been defined by the presence of two central hallmarks, amyloid/senile plaques and neurofibrillary tangles (NFTs), first identified by Alois Alzheimer [65,66]. Extracellular amyloid plaques form from Aβ peptides that oligomerize, aggregate, and associate with other proteins and cellular constituents to form extracellular senile plaques, while intracellular NFTs are formed from the fibrillization of phosphorylated Tau. Aβ is generated by the two-stage hydrolysis of amyloid beta precursor protein (AβPP) by β-secretase (BACE1) and γ-secretase [67]. As another example of the central role of CaM, AβPP, BACE1, and presenilin 1 and 2 (PSEN1, PSEN2), subunits of γ-secretase, are all verified CaMBPs, as are many other critical proteins in neurodegeneration [51,68]. As mentioned above, Aβ also binds CaM in the presence of calcium [41]. NFTs, the second hallmark of AD, can form when Tau phosphorylation displaces pTau from microtubules allowing it to polymerize into tangles in a multistep process. AD has a strong genetic component with mutations in PSEN1 and PSEN2), amyloid beta precursor protein (AβPP), and apolipoprotein E4 (APOE4), causing autosomal dominant early-onset AD, also called familial AD (FAD) [69,70]. Numerous other risk genes (e.g., TREM2, SORL, CLU, CRI, and others) are associated with late-onset forms of the disease [69]. In all AD cases, a diversity of factors (e.g., age, lifestyle, environmental, gender, diabetes, etc.) impact the onset and progression of the disease for which there is no known cure and few therapeutic options for symptomatic relief [70].

In AD, calcium dysregulation has multiple functions, beginning with an initial disruption of cellular metabolism that affects autophagy, neuroinflammation, neuronal repair, and neurotoxicity coupled with the synthesis and aggregation of Aβ and phosphorylation of Tau [71]. It has been established that calcium dysregulation occurs before the appearance of plaques and tangles in cases of mutation-driven FAD. Calcium dysregulation upregulates AβPP cleavage enzymes leading to increased production of Aβ species and increases the enzyme activity of Tau kinases resulting increased pTau levels as a prelude to neurofibrillary tangle formation [46]. Aβ, the hallmark toxic peptide subunit of senile plaques, engages in calcium dysregulation in multiple ways. For example, Aβ oligomers can generate unregulated calcium-permeable pores on the plasma membrane [72]. There is also more recent evidence that Aβo can also induce a rapid calcium ion influx by altering the mechanical properties of the cell membrane, causing a mechanosensitive activation of extrasynaptic NMDAR with a lesser level of activation of AMPAR [73]. There have been reports that Tau can also form ion channels in cellular membranes [74]. As summarized in Figure 2, they each can also bind to and alter the functions of multiple receptors and ion channels, but their combined impact on calcium release by these receptors remains to be fully understood.

Fifty-six percent of AD patients exhibit both Aβ and αSyn pathologies that accelerate neurodegenerative disease progression and reduce survival times. The binding of CaM to toxic aggregates of Aβ and αSyn cannot be ignored as an early and critical event in neurodegeneration. A detailed review of the interaction between these aggregation-prone proteins shows that they act synergistically, generating the amyloid plaques and Lewy bodies that characterize this group of AD patients [75]. This cooperation also appears to extend to interactions of the two toxic proteins with Tau, which has led to additional disease sub-classifications based upon the pathologies associated with them, while the synergistic mechanism remains to be determined. Since Aβ, Tau, and αSyn each individually disrupt calcium signaling in different ways in different cellular subcompartments, a combined effect could explain in part the more rapid neurodegenerative decline observed in individuals with overlapping pathologies.

The usual suspects have been implicated in the disruption of calcium signaling in AD, including those in the cell membrane (e.g., mGluR, NCX, NMDAR, VGCC, and TRPC), ER (IP3R and RyR), and mitochondria (PTP), but not all studies are in agreement as to the relative contributions of each one [46]. Various mGluRs have been implicated in multiple neurodegenerative diseases (e.g., AD, HD, and PD) but mGluR5 is the most well-studied, offering potential as a therapeutic target and diagnostic tool [10]. Studies have implicated mGluR5 in Aβ signaling, underlying toxicity and neuronal death in AD [76]. mGluR5 serves as a receptor for Aβ42 with the binding causing a reduction in mGluR5 lateral diffusion, an increase in receptor clustering, and synaptic localization [27]. These events underlie increased intracellular calcium that causes hyperexcitability, leading to synaptic loss. The interaction of Aβ with other mGluR family members has not been shown. PMCA is a P-type Ca^2+^-ATPase superfamily member that functions in restoring cytosolic calcium levels to nanomolar levels by extruding ions extracellularly [77]. PMCA is activated by CaM binding, an event that enhances calcium efflux [18]. PMCA activity is reduced in AD in part due to the effects of Aβ and Tau, each of which bind to and inhibit its activity, with the level of inhibition varying with the PMCA isoform [29,78].

The calcium channels that have been studied as potential therapeutic symptomatic targets in AD patients and model systems have been reviewed and are listed in Tables 3 and 4 of Guan et al. [79]. They support the general consensus that a limited, but still large, group of cell membrane (i.e., NMDAR, L-VGCC), endoplasmic reticulum (IP3R, RyR1/3), lysosomal (TPC) and mitochondrial (VDAC, mPTP) receptors and channels are critical contributors to calcium dysregulation associated with the disease [46,80]. NMDAR antagonists (e.g., memantine) that reduce calcium influx have shown some success at symptomatic relief in those with moderate to severe AD, but overall, a specific drug to stop calcium dysregulation does not exist [79]. The complex interactions of Aβ with a diversity of identified receptors and channels in the cell membrane, ER, lysosomes, and mitochondria are overwhelming, and a lack of consensus has resulted from studies conducted in different in vitro and in vivo model systems coupled with the use of different Aβ monomers and oligomers. Many of these potential therapeutic targets also bind CaM and other toxic peptides (e.g., Tau, αSyn), adding to this complexity. While various therapeutic approaches are being evaluated by individual research groups, only one phase 3 trial modulating Orai calcium channels and cognition is listed in the 2023 AD drug pipeline [46,81,82].

### 2.2. Huntington’s Disease

HD is a debilitating, hereditary, progressive, and fatal disorder that primarily affects striatal medium-sized spiny neurons (MSNs) and cortical pyramidal neurons (CPNs). The age-dependent, monogenic (*HTT* gene on chromosome 4) neurodegenerative disease can be detected prior to symptom appearance using genetic analysis [83]. Currently incurable, common HD symptoms include cognitive issues, uncontrollable muscle activity, and psychological problems [83,84]. These symptoms result from neuronal atrophy and death, primarily in MSNs and CPNs of the striatum and cerebral cortex. Huntingtin (Htt) protein, encoded by the *HTT* gene, has an extensive polyglutamine (polyQ) repeat that when mutated (mHtt) possesses a longer, more toxic polyQ repeat. Calpain cleaves the protein, releasing fragments that form toxic aggregates, fibrils, and inclusions [85]. Both wild-type Htt and mHtt are experimentally validated calcium-dependent CaMBPs with CaM binding directly related to the length of the polyQ repeat [43]. Compared to wild-type Htt, mHtt binds to CaM with a higher affinity [43]. Htt, CaM, and transglutaminase 2 (TG2), an enzyme that post-translationally modifies Htt, co-localize, and the peptide inhibition of CaM reduces mHtt cytotoxicity and TG2 modification [86,87]. Aggregates of the CaMBP αSyn are also found within Htt inclusions [88].

Extensive evidence suggests that intracellular neuronal calcium signaling and ion channel activity are pivotal to HD progression [6,89,90,91]. For example, Oikonomou et al. studied calcium transients in the R6/2 mouse model of juvenile HD and found that perturbations of calcium homeostasis and recovery occurred in CPNs before and after the onset of overt disease symptoms [91]. Their results also support RyRs and L-type calcium channels as potential therapeutic targets and, in keeping with previous results, support the positive results of treatments with L-type calcium channel blockers such as nifedipine and isradipine. A detailed review of calcium dysregulation in HD by Kolobkova et al. revealed the importance of mHtt in altering the transcription and function of calcium ion channels in the cell membrane, ER, and mitochondria [92]. Also, mHtt alters the levels of mRNA for multiple critical genes encoding proteins involved in intracellular calcium regulation including CaM, calbindin, RyR1, InsP3R1, and subunits of VGCCs [93,94,95].

The mutations in the Htt gene that cause HD affect a number of targets involved in calcium homeostasis, of which upregulating the CaMBP PSEN1 while downregulating CaM could significantly impact downstream calcium signaling [95]. In addition, mHtt binds to IP3R, causing an enhanced release of calcium ions from the ER that in turn enhances calcium entry via SOC [31,95,96]. mHtt binding occurs at the C-terminal cytoplasmic region of IP3R1 [37,63]. The resulting increased calcium generates mitochondrial calcium handling defects, causing increased ROS generation in turn leading to the initiation of apoptosis [97]. Htt also binds to lysosomal membranes where it induces ROS production, impacts autophagy, and affects organelle positioning, but whether this binding generates membrane pores that affect lysosomal calcium levels remains to be seen [98]. According to the Huntington’s Disease Society of America website (https://hdsa.org/hd-research/therapies-in-pipeline/, accessed on 8 October 2023), two NMDA receptor antagonist studies are in 2023 phase 2 HD trials, but no other calcium dysregulation studies are apparently underway.

### 2.3. Parkinson’s Disease

The second most common neurodegenerative motor disease, PD is characterized by the loss of dopaminergic (DA) neurons, decreased dopamine levels, and the presence of intraneuronal αSyn-rich Lewy bodies (LBs) [99]. Increasing exponentially after age 65, PD is a slow, progressive motor disease characterized by shaking, stiffness, and difficulties with coordination and balance due to the degeneration and death of DA neurons in the substantia nigra pars compacta [100,101,102]. Those with the disease also suffer mild cognitive impairment that progresses to dementia over time. The D2-dopamine receptor (D2DR), which mediates the actions of dopamine, binds to and is inhibited by CaM, but how this applies to PD symptomology remains to be clarified [103].

Along with αSyn, LBs commonly contain parkin, neurofilaments, and ubiquitin, but over 200 other proteins and cellular constituents have been found within them, varying with the type and location of LBs [104]. PD differs from other forms of Lewy body dementia (LBD) based on the primary localization of LBs in DA neurons from the substantia nigra pars compacta region. While αSyn is widely used as a diagnostic tool and biomarker for PD, Aβ, Tau, and αSyn are also found in LBs in both PD and LBD [105]. In PD, LBD, and other synucleinopathies, CaM can bind to αSyn in a calcium-dependent manner, enhancing fibril formation as a prelude to LB formation [106]. In addition, the aggregation of αSyn to form fibrils leads to calcium dyshomeostasis disrupting downstream CaM-mediated signaling events [107,108].

One of the early stages on the road to PD-based neurodegeneration is the dysregulation of intracellular calcium levels. In support of this, the treatment of primary neuronal cultures with recombinant αSyn monomers or oligomers causes an increase in cytosolic calcium [109]. An overload of cytosolic calcium plays a significant role in the events of PD by causing excitotoxicity that can cause DA neuron neurodegeneration and death [9,110,111]. The disruption of cytosolic calcium homeostasis involves ion channels in the cell membrane as well as calcium release from the ER, mitochondria, and lysosomes [102]. Transient increases in intracellular calcium have been shown to generate an increase in αSyn aggregates in human cell lines expressing the toxic protein [112].

While increased calcium levels augment αSyn aggregation, the resultant αSyn oligomers also feed back to contribute to calcium dysregulation by affecting surface receptors (e.g., adenosine receptor) and channels (NMDAR, mGluR) [113]. αSyn binds to the N-terminal of mGluR5, an event that stimulates neuroinflammation in PD [28]. αSyn aggregates also bind to and activate SERCA, contributing to extra cytosolic calcium [114]. In addition to stimulating αSyn aggregation, calcium dysregulation drives αSyn secretion from neurons, where its accumulation interstitially is followed by its uptake by other neurons that, in a vicious cycle, further promotes increased calcium levels [114,115]. Additionally, αSyn, which can bind to a diversity of membrane types, may form cell membrane channels allowing unregulated calcium influx [109,114].

It is clear that there is little consensus among the experts about the causes of PD, which include αSyn alone or in combination with a long list of other factors such as mitochondrial dysfunction, increased leucine-rich repeat kinase 2 (LRRK2, PARK8, and dardarin) activity, exposure to toxins and various combinations of biological, environmental, genetic, gut microbiome, and social factors, among others [116]. Genetic mutations in certain genes (e.g., PINK1 and PRKN) are extremely strong predisposing risk factors for PD, while others (e.g., glucocerebrosidase (GBA) and LRRK2) can increase one’s risk [117,118]. Mutations in the PARK7 gene cause early-onset, autosomal recessive PD [119]. That said, genetics only explains about one-third of disease risk [120]. While their specific impact is still under study, aging, environmental factors, lifestyle, and other factors can modify the impact of disease mutations. One significant environmental factor is pesticides, especially paraquat, rotenone, and organochlorines, which have been shown to cause PD symptoms regardless of genetic makeup [121].

Pesticides are known to alter calcium homeostasis by affecting various channels and receptors, in turn causing a harmful cytosolic increase in calcium levels that underlies neuronal dysfunction and several neurodegenerative diseases [122]. With around 300,000 individuals killed by pesticides each year and their use continuing to increase with agricultural industrialization, they present a serious health risk worldwide, but especially in poorer and developing countries [122]. Pesticides of different chemical classes have been shown to cause calcium dyshomeostasis with the calcium overload driving oxidative stress, neuroinflammation, and neuronal death, events involved in the development of PD, AD, HD, and other neurodegenerative diseases [122,123,124]. Pesticides allow the entry of calcium ions through cell membrane channels (esp. VGCCs, PMCA) and from the endoplasmic reticulum. The elevated calcium levels are also sustained due to the inhibition of key enzymes involved in calcium homeostasis [122]. For example, exposure to rotenone induces kinase activation that can phosphorylate PMCA and VGCCs to promote calcium influx. In addition, it has long been known that certain pesticides (e.g., permethrin and cypermethrin) are potent inhibitors of the phosphatase PP2B. Since PP2B participates in calcium channel regulation and, along with CaMKII, is linked to learning and memory, the negative impact on neuronal function is significant.

Bohush et al. reviewed a handful of the CaMBPs that are associated with PD (i.e., CaMKII, PP2B, NMDAR, AchR, Adenosine A2AR, and cdk5) but did not cover other critical CaMBPs discussed above (e.g., αSyn, Aβ, Tau, and D2DR) [125]. While those covered here relate to calcium dysregulation, αSyn function, and LB formation, to set the record straight, several other CaMBPs involved in PD also deserve mention. The CaMBP Ng is not only a biomarker for PD but also for AD and Creutzfeldt-Jakob disease [126]. Mutations in the GBA gene (*GBA1*) result in an increased risk of PD and other synucleinopathies [127]. While both multifunctional enzymes are involved in multiple neurodegenerative events, substrates for TGM2 include Aβ, Tau, α-Syn, and mHtt, thus affecting the toxic potential of these classic marker proteins [128]. While their binding remains to be verified experimentally, other key marker and risk proteins for PD possess one or more canonical binding domains, indicating they are potential CaMBPs: PINK1, LRR2, and PARK7 [129].

## 3. Specific Ion Channels and Receptors Linked to Calcium Dysregulation

### 3.1. VGCC Families Involved in Neurodegeneration

Voltage-gated calcium channels (VGCCs, CaV) are important in calcium-signaling events central to models of learning, memory, and plasticity [130,131]. CaM binding is a highly conserved attribute of all VGCCs occurring via a calcium-independent C-terminal IQ motif (I/L/V)QXXXRXXXX(R/K) and a calcium-dependent N-terminal (xWxxx(I or L)xxxx) [19,30]. CaM controls the calcium influx through VGCCs via calcium-dependent inactivation (CDI) and calcium-dependent facilitation (CDF), as detailed elsewhere [132,133]. Based on their biophysical and pharmacologic attributes, VGCCs are classified as L-, N-, P/Q-, or N-type, with a total of 10 subfamilies: L-type (CaV1.1, CaV1.2, CaV1.3, and CaV1.4), P/Q-type (CaV2.1), N-type (CaV2.2 and NTCC), and R-type (CaV2.3) [134]. The Cav subfamily members display different primary tissue-specific localizations including those found in the cell bodies and dendrites of neurons: Cav1.2, Cav1.3, Cav2.1, Cav2.2, Cav2.3, Cav3.1, Cav3.2, and Cav3.3. In presynaptic neurons, calcium influx through Cav2.1 and Cav2.2 channels stimulates synaptic vesicle exocytosis [135]. The improper regulation of Cav2.2 is involved in HD, multiple sclerosis (MS), and other neurodegenerative diseases [134]. For example, normal and mHtt each bind to Cav2.2, causing an increase in the activity of this N-type VGCC [31,95].

Cav1.2 of the Cav1 subfamily, which makes up 90% of the L-type calcium channel (LTCC) members in the CNS, is of special interest in PD [102]. LTCCs are expressed in dopaminergic neurons that, due to excitotoxicity caused by dysregulated calcium levels, degenerate in this disease [111]. In healthy neurons, the pace-making activity of Cav1.2 and Cav1.3 VGCCs regulate dopamine (DA) release [136]. Neurodegeneration in PD occurs predominantly in SNc dopaminergic neurons that mainly rely on Cav1.3 L-type VGCCs for their basal activity. Increased expression of Cav1.3 occurs in PD individuals, resulting in increased cytosolic calcium, mitochondrial stress, and, over time, neuronal death [137,138]. At present, no selective or effective Cav1.3 inhibitors exist. On the other hand, there was some early success with L-type channel blockers since patients treated with dihydropyridine channel blockers had a reduced incidence of PD compared to those treated with other antihypertensive drugs [139]. However, a recent re-analysis of 20 studies concluded that there was only weak evidence that antihypertensive drugs had any value in treating PD [140]. There is some hope that selective phytochemicals from medical plants that reduce hypertension might be a therapeutic option for treating neurodegenerative disorders, but this area needs to be actively studied [102]. Despite this, there is little focus on calcium channels in current drug trials since only one 2023 PD drug trial focuses on an inhibitor of T-type calcium channels [141]. On the other hand, 14 total drug trials on αSyn, spread across all phases, are currently underway, some of which could provide insight into the impact of this toxic biomarker on calcium channels.

### 3.2. LRRK2: A VGCC Regulator

A member of the Ras of complex proteins family, LRRK2 is a serine/threonine kinase with a Ras-like GTPase domain. Early work showed that LRRK2 increases PD risk in both sporadic and dominantly inherited forms of the disease, and patients with genetic links to the disease appear to be clinically indistinguishable from those with idiopathic late-onset PD [142]. LRRK2 is involved in calcium signaling, buffering, and homeostasis, at least in part through its regulation of VGCCs [143]. While its complete modes of action are under study, LRRK2 has been shown to act directly on L-type VGCCs (e.g., Cav2.1). Over a dozen mutations in LRRK2 cause an increased risk for PD, but disease penetrance varies widely from ~17 to 80% [144]. Studies on these mutations have generated a deeper understanding of the various PD subgroups that differ from sporadic and inherited forms of the disease. LRRK2 mutations typically cause an increase in its kinase activity and, as a result, LRRK2 kinase inhibitors are undergoing human clinical trials [141]. It is important to note that LRRK2 is a risk factor, not a primary cause of PD, and, according to Taymans et al., “the end goal of validating LRRK2 as a PD therapeutic target has yet to be reached” [144]. As part of a study of CaMBPs involved in neuroinflammation, LRRK2 is a potential CaMBP because it contains a canonical 20 aa CaMBD at position 776–795, within which four binding motifs are present [129]. Validating CaM binding would open up a whole new area for the regulation of LRRK2 and its potential functions in PD and other diseases.

### 3.3. Endoplasmic Reticulum Calcium Homeostasis and Dysregulation

The ER is the primary intracellular calcium storage site and IP3R, RyR, and SERCA are the most studied ER components involved in calcium homeostasis and dysregulation. IP3R and RyR release calcium into the cytosol while SERCA uptakes calcium to re-establish ER calcium homeostasis. Early work showed that FAD mutations drive aberrant calcium release from the ER via IP3R and RyR channels [145,146,147]. Since then, more insight into the complexity of the cellular components involved in ER calcium homeostasis and dysregulation has been revealed, some of which is summarized in Figure 2. When activated, the glutamic acid receptor mGluR directs the generation of inositol 3 phosphate (IP3) that activates IP3R to release calcium into the cytosol. Another ER receptor involved in calcium efflux, RyR2 is involved in calcium dyshomeostasis in AD as well as PD [148,149,150].

mGluR, IP3R, and RyR are CaMBPs. CaM binds to the cytoplasmic tail of mGluR where it functions in receptor translocation [16]. All IP3R subtypes bind to CaM, an event that inhibits receptor function [24]. RyRs possess three CaMBDs involved in receptor function [24,25]. The large tetrameric RyR2 is a CaM-binding protein [149,151]. The dissociation of CaM from RyR2 plays a crucial role in the pathogenesis of AD, not only due to ER stress effects but also by contributing to toxic cytosolic increases in calcium levels. In an AD mouse model, the dissociation of CaM from RyR2 caused calcium release, contributing to events underlying neuronal apoptosis and cognitive dysfunction [149]. To date, no studies have researched the combined interactive significance of this binding and regulation.

The continued efflux of calcium ions can lead to depleted ER calcium levels. SERCA, a member of the mammalian P-type ATPase superfamily, is a Ca^2+^-ATPase that functions in restoring ER calcium levels [77]. While SERCA does not bind CaM, it is phosphorylated (Ser16) by CaMKII, which enhances ion uptake into the ER [152,153]. SERCA is a store-operated calcium channel (SOC) involved in regulating calcium entry (SOCE). It is also recognized as a calcium-release-activated calcium (CRAC) channel since it is a SOC consisting of the Orai ion channel and stromal interaction molecule (STIM) protein families [154,155]. Thus, this regulatory interaction involves events operating at both the ER and cell membrane. STIM is a calcium sensor localized in the ER [156,157,158]. The structures of STIM1 and 2 have been detailed, but only certain aspects are examined here [155]. At homeostatic calcium levels, due to calcium binding to EF-hand domains on the N-terminal of STIM, the protein has a compact structure tightly associated with the ER with its Orai-activating region unavailable for interaction with the ion channel [155]. When ER calcium stores become depleted, the loss of EF-hand calcium induces a conformational change in STIM, revealing the Orai-interacting region. STIM oligomerization and translocation to ER–cell membrane junctions allows STIM to interact with Orai, causing channel opening for calcium influx from the extracellular environment [155]. Calcium-activated CaM binds to STIM near the C-terminal, disrupting the STIM/Orai interaction and shutting down calcium influx [33]. CaM also binds to the N-terminal of Orai proteins where it is involved in CDI of SOCE [21,159]. Orai/STIM SOCE is involved in numerous age-related neurodegenerative diseases including AD, HD, and PD [155,159]. Since CaM mediates several events in Orai/STIM SOCE and since toxic biomarkers (i.e., Aβ, Tau, Htt, and αSyn) each bind to CaM, there are potentially multiple levels of calcium regulation in neurodegeneration that remain to be studied.

### 3.4. Mitochondrial Calcium Dysregulation

Calcium regulation in mitochondria is summarized in Figure 2, but due to the double-membrane construction of this organelle, some additional points are addressed here. Mitochondria possess stores of calcium that contribute to cytosolic calcium levels. Disturbances in calcium homeostasis within them are critical to neuronal function and survival [160]. Disrupting calcium levels in mitochondria can lead to malfunction of the electron transport chain (ETC), resulting in decreased ATP generation and increased ROS formation, causing neuron death [161]. The mitochondrial cascade hypothesis of AD proposes that early mitochondrial dysfunction involves a series of events: decreased metabolism involving disrupted Ca^2+^ homeostasis that leads to increased ROS, and subsequent events resulting in the activation of pro-apoptosis [162].

The predominant mitochondrial membrane protein, voltage-dependent anion channel 1 (VDAC1) is one of three VDAC family members. It serves a central role in mitochondrial Ca^2+^ homeostasis and mediates crosstalk between mitochondria, the ER, and other cellular constituents that regulate mitochondrial structure and function [163]. VDAC1 is implicated in AD because not only is it expressed at high levels in AD brains, but it also directly interacts with Aβ and pTau (Figure 2) [34,164,165]. In addition, VDAC1 mediates Aβ entry into the cell, events that lead to mitochondrial dysregulation and apoptosis. Aβ has also been shown to induce VDAC1 expression and cell death in a FAD mouse model, in which primary neuronal cell cultures were treated with it [165]. VDAC1 is a CaMBP, and CaM binding modulates VDAC1 gating and permeability as well as significantly reduces its conductivity [23]. αSyn also binds to and regulates VDAC function, but its interactions and effects are much more complex [36]. αSyn translocation into the mitochondrial matrix targets specific proteins (e.g., mitochondrial permeability transition pore, mPTP) and the retention of αSyn within the VDAC channel causes a major increase in calcium ion permeability [36,166]. Each of these effects of αSyn has implications beyond the focus of this review. While not as well-studied, a single abstract has indicated that Htt directly interacts with human and yeast VDAC, causing a strong effect on channel conductance in yeast [35]. Thus, VDAC1 presents another example of a critical channel involved in calcium homeostasis that not only binds to and is regulated by CaM, but it also binds to and is regulated by the toxic peptides Aβ, pTau, Htt, and αSyn.

### 3.5. Lysosomal Calcium and Neurodegeneration

Containing around half the calcium concentration of the ER, lysosomes are potentially a significant contributor to calcium homeostasis and dyshomeostasis (Figure 2). A large number of proteins that affect lysosomal function are involved in several sporadic neurodegenerative diseases including AD, ALS, FTD, and PD. Some of these are known or presumptive CaMBPs, as discussed above. The CaMBPs GBA1, αSyn, PSEN1/2, and Tau regulate various lysosomal functions, while others including αSyn participate in autophagy [167]. The importance of lysosome function in lysosomal storage diseases (e.g., Tay Sachs disease, Neimann-Pick disease) and autophagy have long been studied, but its importance in other neurodegenerative diseases is still being revealed [168]. In addition to digesting and processing a diversity of macromolecules, lysosomes are also storage depots for cytosolic calcium ions [167,169]. Decreased levels of lysosomal calcium have been reported in cases of familial AD caused by mutations in the CaMBPs PSEN1/2 and AβPP [170]. In PD, the presumptive CaMBP LRRK2 and validated CaMBP GBA are both involved in lysosomal calcium release [168]. Glucocerebrosidase (GBA1), a genetic risk factor for PD and a cause of Gaucher disease, leads to impaired calcium release from lysosomes, possibly due to reduced levels of stored calcium [171].

Flux across the lysosomal membrane occurs via a small group of channels: P2X4 (an ionotropic P2X family receptor member), TPCs (two-pore channels), TRPA1 (non-selective ion channel), TRPMLs (TRP ion channel family), TRPM2 (a member of the melastatin group of the TRP family), and VGCCs (P/Q-type voltage-gated channels). The importance of these channels in neurodegeneration has been reviewed [168,172,173]. Mammals have 28 cationic channel transient receptor potential (TRP) ion channel superfamily members that are classified into 6 subfamilies, of which TRPC (canonical) and TRPML (mucolipin) are of interest here [26]. All TRPCs are CaMBPs whose functions are linked to neurocytotoxic events underlying neurodegeneration [20]. The role of CaM as a regulator of calcium homeostasis and dyshomeostasis in lysosomes is not well-understood. Lysosomes bind to CaM, which mediates membrane fusion events driven by lysosomal calcium [174]. For example, TRPC4 binds to CaM in the presence of calcium, an event that inhibits channel function [32]. IP3R competes for the CaM-binding site, displacing apoCaM and restoring TRPC channel function when calcium levels drop. This “conformational coupling” event via the CaM/IP3R (TRP motif)-binding region is a main regulatory gating mechanism for SOCs [175]. TRPML1, which is permeable to a number of monovalent and divalent cations including H^+^, K^+^, Na^+^, Fe^2+^, Mn^2+^, and Zn^2+^, localizes to lysosomes and endosomes and contains the same regulatory TRP motif [26,174]. TRPML1 has been implicated in several neurodegenerative diseases: AD, PD, ALS, and Niemann-Pick [172]. The loss of function of TRPML1 is involved in neurodegeneration including APOE-linked late onset AD [176].

## 4. Conclusions

One of the earliest events linked to most, if not all, neurodegenerative diseases is the dysregulation of calcium. Dozens of buffering proteins, ion channels, sensors/effectors and receptors are involved in calcium homeostasis and neurodegenerative dysregulation (Figure 2). A primary target of calcium signaling is CaM, and the diverse and critical protein ecosystem mediated by CaM signaling in calcium dysregulation is extensive. Previously, the function of other CaMBPs (e.g., CaMKII, calcineurin) affected by calcium dysregulation in neurodegenerative events have been reviewed [133]. Here, we have seen that CaM binding also regulates multiple cell membrane channels and receptors (mGluR, NMDAR, PMCA, VGCC, TRPC, Orai1/2, and NCX) as well as those in the ER (IP3R and RyR), mitochondria (NCX and VDAC), and lysosomes (VGCC and TRPML1) (Figure 2). The role of CaM in regulating calcium dyshomeostasis in neurodegeneration provides more support for the universality of the Calmodulin Hypothesis [68,177]. Added to this, many of these CaM-binding events are coupled with the binding of toxic protein disease biomarkers to specific receptors and calcium ion channels: mGluR (Aβ; αSyn), NMDAR (Aβ; αSyn), PMCA (Aβ), VGCC (Aβ; Htt), SOC (Aβ) NCX (Aβ; Tau), IP3R (Tau; Htt; αSyn), RyR (Aβ; Tau), and VDAC (Tau). Aβ, Tau, mHtt, and αSyn are all calcium-dependent CaMBPs [41,42,43,44]. The complex interplay between CaM, receptors, and channels involved in calcium regulation and toxic biomarkers is only now coming to light.

While a great deal is known about the role of CaM in regulating primary calcium ion channel families, this knowledge is still insufficient since their coregulation by toxic peptides (i.e., Aβ, Tau, Htt, and αSyn) and other agents remain to be fully investigated. This suggests that current attempts to target them to control calcium levels are fraught with problems. Since multi-pathologies are common in neurodegenerative diseases, varying numbers of toxic peptides could operate together to affect the receptors and ion channels involved in calcium regulation. A review of Figure 2 will bring to light this potential complexity. Based on this, therapeutic targeting of receptors and channels involved in calcium regulation might not offer the specificity required to be effective. The apparent waning of interest in targeting calcium channels might also be signaled by the limited number of calcium-based studies that are currently in the AD, HD, and PD drug pipelines.

## Data Availability

Not applicable.

## Abbreviations

AchR	acetylcholine receptor
AD	Alzheimer’s disease
ALS	amyotrophic lateral sclerosis
AMPAR	α-amino-3-hydroxy-5-methyl-4-isoxazolepropionic acid receptor
APOE	apolipoprotein E
aSyn	alpha-synuclein
Ca^2+^	calcium cations
[Ca^2+^]i	intracellular free Ca^2+^ concentration
CaM	calmodulin
CaMBD	calmodulin-binding domain
CaMBP	calmodulin-binding protein
CaMKII	Calmodulin-dependent protein kinase II
CaV	Voltage-gated Ca^2+^ channels
cdk5	cyclin-dependent kinase 5
CLU	clusterin
D2DR	D2-dopamine receptor
ER	endoplasmic reticulum
FTD	frontotemporal dementia
GBA	glucocerebrosidase
GPCR	G-protein-coupled receptors
HD	Huntington’s disease
IP3R	inositol (1,4,5)-trisphosphate receptor
KCa	Ca^2+^-activated K^+^ channels
LB	Lewy body
LBD	Lewy body dementia
LRRK2	leucine-rich repeat kinase 2
MCU	mitochondrial Ca^2+^ uniporter
mGluR	metabotropic glutamate receptor
mPTP	mitochondrial permeability transition pore
MS	multiple sclerosis
NCX	Na^+^/Ca^2+^ exchanger
NFTs	neurofibrillary tangles
Ng	neurogranin
NMDAR	N-methyl-D-aspartate receptor
Orai1/2	Calcium-release-activated calcium channel protein 1 and 2
PD	Parkinson’s disease
PARK7,8	Parkinsonism-associated deglycase 7,8
PILRA	paired immunoglobin-like type 2 receptor alpha
PINK1	PTEN-induced kinase 1
PMCA	plasma-membrane Ca^2+^-ATPase
PP2B	protein phosphatase 2B; calcineurin
ROC	receptor-operated channels
ROS	reactive oxygen species
RyR	ryanodine receptor
SERCA	sarcoplasmic reticulum Ca^2+^-ATPase
SNCA	a-synuclein
SOC	store-operated calcium channel
SOCE	store-operated calcium entry
STIM	stromal interacting molecule
TGM1/2	transglutamase 1/2
TREM2	triggering receptor expressed on myeloid cells 2
TRPC	transient receptor potential channel
TRPML	TRP ion channel family
VDAC	voltage-dependent anion channel 1
VGCC	voltage-gated calcium channel.
